# Exercise Mediates Noncoding RNAs in Cardiovascular Diseases: Pathophysiological Roles and Clinical Application

**DOI:** 10.1017/erm.2024.25

**Published:** 2024-11-21

**Authors:** Changyong Wu, Xiaocui Chen, Lu Yang, Huang Sun, Suli Bao, Haojie Li, Lihui Zheng, Huiling Zeng, Ruijie Li, Yunzhu Peng

**Affiliations:** 1 Department of Cardiology, The First Affiliated Hospital of Kunming Medical University, Kunming, Yunnan, China; 2 Department of Gastroenterology, Affiliated Hospital of Panzhihua University, Panzhihua, Sichuan, China

**Keywords:** cardiovascular disease, cardiac fibrosis, cardiac rehabilitation, exercise, molecular mechanism, noncoding RNA

## Abstract

Exercise-based cardiac rehabilitation is effective in improving cardiovascular disease risk factor management, cardiopulmonary function, and quality of life. However, the precise mechanisms underlying exercise-induced cardioprotection remain elusive. Recent studies have shed light on the beneficial functions of noncoding RNAs in either exercise or illness models, but only a limited number of noncoding RNAs have been studied in both contexts. Hence, the present study aimed to elucidate the pathophysiological implications and molecular mechanisms underlying the association among exercise, noncoding RNAs, and cardiovascular diseases. Additionally, the present study analysed the most effective and personalized exercise prescription, serving as a valuable reference for guiding the clinical implementation of cardiac rehabilitation in patients with cardiovascular diseases.

## Introduction

Cardiovascular diseases (CVDs) have remained the most common cause of morbidity and mortality worldwide for over a decade, suggesting that innovative approaches are urgently needed to fight these major public health problems (Ref. 1). Both the guidelines on the secondary prevention of CVDs and cardiac rehabilitation (CR) state that exercise, as a central element, is an effective nonpharmacological and nontraumatic intervention (Refs. 2–4). Increasing studies have shown that regular exercise training (ET) reduces cardiovascular risk factors, including the promotion of weight loss, control of blood pressure, improvement of hyperlipidaemia, and insulin sensitivity (Refs. 5–7. Moreover, exercise-based CR is effective in improving exercise capacity, cardiopulmonary function, cardiovascular symptoms, adverse events, and quality of life in patients with hypertension, coronary heart disease (CHD), valvular heart disease, and heart failure (HF) (Refs. 8–10). Although strong and compelling evidence supporting the benefits of exercise has increased over the past years, its complex molecular mechanisms remain obscure.

Increasing research interest has been focused on the pathological processes of exercise-induced cardioprotection (EIC), including the inflammatory response, myocardial oxidative stress, cardiac hypertrophy, vascular remodelling, myocardial metabolic adaptations in mitochondrial function and glucose/lipid metabolism, and systemic responses (Ref. 11). From a molecular standpoint, there is a limited understanding of the signalling pathways responsible for these processes. Similarly, translating insights from CVD research into clinical applications, such as warnings and controls of exercise risk, has been challenging. Noncoding RNAs (ncRNAs) have been recognized as a regulatory network governing gene expression in multiple pathophysiological processes, such as epigenetic, transcriptional, and posttranscriptional levels (Refs. 12, 13). However, little is known about the expression and function of these ncRNAs in response to exercise and how they benefit cardiovascular health.

The presented review aimed to summarize the most recent publications on the pathophysiological mechanism of ncRNAs in EIC, discuss potential therapeutic strategies and propose considerations regarding the present and future of research in this field.

### Beneficial effects of ET for cardiovascular diseases

**Figure 1.**
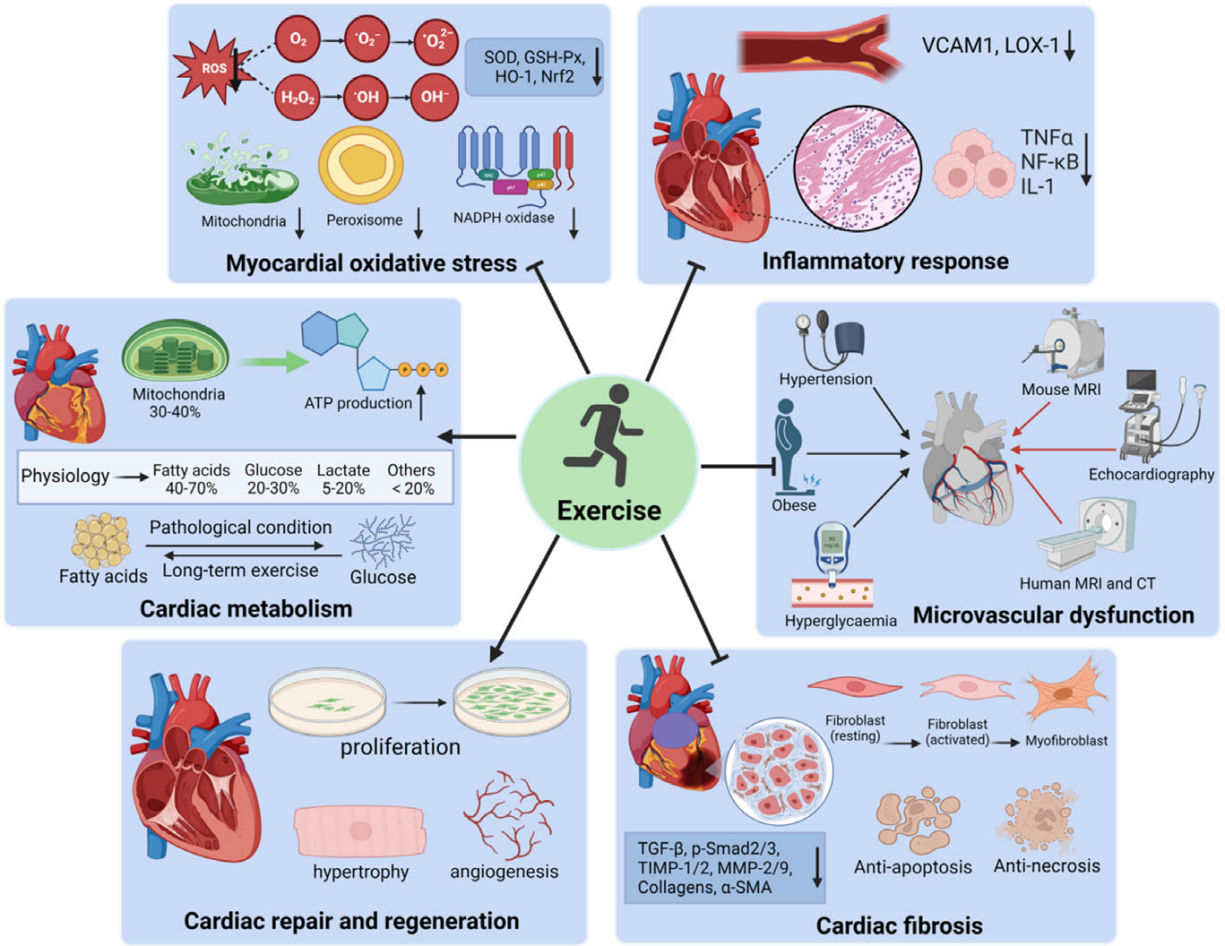
Protective effects of exercise rehabilitation in CVDs. Exercise-based cardiac rehabilitation plays a significant role in the pathophysiological evolution of cardiovascular health, including reducing myocardial oxidative stress and the inflammatory response, improving microvascular dysfunction and cardiac fibrosis, and promoting cardiac metabolism, physiological hypertrophy and cardiomyocyte proliferation. These benefits may reduce the incidence of cardiovascular complications, the rehospitalization rate, and mortality. VCAM1, vascular cell adhesion molecule-1; LOX-1, lectin-like oxidized LDL-receptor-1.

An accumulating number of cohort studies, systematic reviews, and meta-analyses have documented that ET has multiple beneficial effects for patients with CVDs, such as improving cardiac structure and function, reducing hospitalization, and all-cause mortality, extending life expectancy (Refs.^14^–^16^). A meta-analysis has suggested that achieving the recommended physical activity levels (150 minutes of moderate-intensity aerobic activity per week) can reduce CVD incidence by 17%, CVD mortality by 23%, and type 2 diabetes mellitus (T2DM) incidence by 26% (Ref.^17^). Compared to an unhealthy diet and inactivity (UDI), a prospective cohort study has revealed that the reduction in all-cause and CVD mortality is associated with the following lifestyles: a healthy diet and activity (HDA); a healthy diet but inactivity (HDI); an unhealthy diet but activity (UDA) (Ref. 18). However, Kivimäki and colleagues (Ref. 19) demonstrated that physical inactivity is associated with a 24% high risk of CHD, a 16% enhanced risk of stroke, and a 42% higher risk of T2DM. The frequency of adverse cardiovascular events in acute endurance runners is equivalent to that in a population with a diagnosis of CHD (Ref. 20). Extreme endurance exercise may induce adverse cardiorenal interactions (Ref. 21). Currently, ET has been prescribed as a medical therapy for different CVDs. In the following section, we will discuss the potential protective effects of ET in CVDs in detail (Figure 1).

### ET reduces myocardial oxidative stress

Reactive oxygen species (ROS), both as subcellular messengers in signal transduction pathways and as contributors to oxidative stress, play beneficial or deleterious roles in the initiation, development, and outcomes of CVDs. The main sources of ROS at the cardiac level are the mitochondrial electron transport chain, xanthine oxidase, NADPH oxidases (more specifically that of NOX5), and nitric oxide (NO) synthase (Ref. 22). Under physiological conditions, producing a low level of ROS is equivalent to detoxification, and it plays a pivotal role in cellular signalling and function. This process, termed redox signalling, is defined as the specific and reversible oxidation/reduction modification of cellular signalling components for the regulation of gene expression, excitation-contraction coupling, cell growth, migration, differentiation, and death (Ref. 22). In contrast, in pathological situations, ROS causes oxidative modification of major cellular macromolecules (such as lipids, proteins, or DNA) in subcellular organelles, including mitochondria, the sarcoplasmic reticulum, and the nucleus. However, this response leads to atherosclerosis, endothelial and mitochondrial dysfunction, increased blood pressure, and cardiomyocyte hypertrophy (Ref. 23).

Not surprisingly, numerous randomized clinical trials investigating antioxidants have been negative, whereas targeting mitochondria will be a promising strategy to improve mitochondrial functionality by nonpharmacological methods, including exercise. In the past few decades, ET has been developed into an established evidence-based treatment strategy for CVDs (Refs. 8, 24). A previous study in a mouse model has demonstrated that ET increases endothelial sirtuin 1 (SIRT1) levels and reduces the downregulation of superoxide dismutase (SOD), glutathione peroxidase (GSH-Px), nuclear factor erythroid 2-related factor (NRF2), and heme oxygenase 1 (HO-1). ET protects against cardiac damage by inducing hyperlipidaemia-induced oxidative stress, inflammation, and apoptosis (Ref. 25). Additional studies in patients with CHD have demonstrated that regular moderate ET attenuates cardiac oxidative stress by decreasing protein carbonyl, SOD, and GSH-Px, as well as increasing GSH and ferric reducing antioxidant power (FRAP) levels (Refs. 26, 27). Additionally, oxidative stress is associated with the expression of ncRNAs (Refs. 28, 29). Masoumi-Ardakani and colleagues (Ref. 30) elucidated that ET improves cardiac function in hypertensive individuals by increasing total serum antioxidant capacity, which is related to reduced microRNA-21 (miR-21) and miR-222 levels. Knockdown of lncRNA SNHG8 in MI mice reduces myocardial infarction (MI) size and alleviates myocardial tissue injury and oxidative stress (Ref. 31). Similarly, lncRNA NORAD overexpression attenuates doxorubicin (DOX)-induced cardiac pathological lesions by decreasing cardiomyocyte apoptosis and mitochondrial ROS levels (Ref. 32). However, further mechanisms of ROS and ncRNAs in ET-induced CVDs remain to be understood.

### ET blunts inflammatory pathways

The inflammatory response is an equivocal topic in cardiovascular protection. It is well known that inflammation is involved in the development and progression of CVDs, such as atherosclerosis, hypertension, CHD, and rheumatic heart disease, and it provokes cardiomyocyte damage. Importantly, ET halts the action of inflammatory mediators and the induction of biological pathways to protect the heart (Refs. 33–35). For instance, ET affects macrophage function, including downregulation of the interleukin-1 (IL-1), tumour necrosis factor-α (TNF-α), and nuclear factor (NF)-κB inflammatory cytokines, as well as reducing oxidized LDL and improving antioxidant capacity to blunt the processes of atherosclerosis (Ref. 33). Evidence from a human study shows that aerobic training is beneficial for blood pressure control and CVD risk reduction by decreasing endothelin-1 and the C-reactive protein (CRP), monocyte chemoattractant protein-1, vascular cell adhesion molecule-1, and lectin-like oxidized LDL-receptor-1 inflammatory markers (Ref. 36). A systemic review and meta-analysis have demonstrated that ET reduces CRP, fibrinogen, and von Willebrand factor (vWF) concentrations in CHD subjects (Ref. 37). Nonetheless, further animal and clinical studies with high methodological qualities and large sample sizes are needed to improve evidence-based medicine in this area and to explore the underlying molecular mechanism of different exercise mode-induced inflammation reduction in more CVDs.

NcRNAs play regulatory roles in inflammation and innate immune responses. The expression of lncRNA INKILN is downregulated in contractile vascular smooth muscle cells (VSMCs) but induced in human atherosclerotic vascular diseases (ASVDs) and aortic aneurysms. Knockdown of lncRNA INKILN by siRNA attenuates the expression of a series of proinflammatory genes by blocking IL-1β-induced nuclear localization and the physical interaction between p65 and megakaryocytic leukaemia 1 (MKL1), which is a major transcriptional activator of vascular inflammation (Ref. 38). MiR-15a-5p and miR-199a-3p overexpression decreases inflammatory pathway protein levels, such as IKKα, IKKβ, and p65, and it reduces oxidized LDL and NF-κB activation in VSMCs and patients with atherosclerosis (Ref. 39). Moreover, overexpression of miR-340-5p reduces cardiomyocyte apoptosis and inflammation via the HMGB1/TLR4/ NF-κB pathway in myocardial ischemia–reperfusion injury (MIRI) (Ref. 40).

### ET optimizes cardiac metabolism

Cardiac metabolism represents a crucial and significant bridge between health and CVDs. The predisposing factors of CVDs, such as insulin resistance, DM, and obesity, are associated with imbalances in cardiac mitochondrial dynamics, mitochondrial fusion and fission, mitochondrial metabolic dysfunction, and mitophagy (Refs. 41, 42). The heart is an important energy-consuming organ, accounting for approximately 30%–40% of mitochondria by volume of cardiomyocytes. Under physiological baseline conditions, the heart is considered an omnivore organ because it uses broad energy substrates. Cardiac ATP is mainly produced from fatty acid oxidative phosphorylationglucose oxidative phosphorylationlactateand other energy sources, including pyruvate, pyruvate, acetate, and branched-chain amino acids (BCAAs). However, under pathological conditions, such as MI, myocarditis, and HF, cardiometabolic substrates switch from major fatty acid oxidation to carbohydrate oxidation (Ref. 43), because glycolysis consumes less oxygen than fatty acid oxidation and the oxidative phosphorylation products, namely, water and carbon dioxide, are nontoxic to the heart.

Compared to pathological conditions, cardiac workload and myocardial oxygen consumption markedly increase during ET, leading to an increased rate of ATP generation by increasing the use of fatty acid and lactic acid, as well as by reducing the consumption of glucose. However, myocardial glucose is significantly used during long-term exercise, which is related to the progression of cardiac physiological hypertrophy (Refs. 44, 45). A previous study has demonstrated that swimming exercise-induced adaptation leads to cardiac physiological hypertrophy and increases glycolysis, glucose oxidation, fatty acid oxidation, and ATP production (Ref. 46). In addition, ET improves the imbalance of mitochondrial dynamics and abnormal changes in mitochondrial structure in pathological states. Treadmill exercise has been identified to attenuate DM-induced cardiac dysfunction by enhancing the cardiac action of fibroblast growth factor 21 (FGF21) and inducing the AMPK/FOXO3/SIRT3 signalling axis to prevent toxic lipid-induced mitochondrial dysfunction and oxidative stress (Ref. 47). A previous study involving ET in a mouse model has reported that ET activates the SIRT1/PGC-1α/PI3K/Akt signalling pathway to attenuate MI-induced mitochondrial damage and oxidative stress (Ref. 48). However, the biological mechanism of ET intervention to improve the energy metabolism of CVDs still lacks a theoretical basis and practical measures.

### ET improves microcirculation structure and function

Recent invasive investigations have shown that nearly 60% patients of chest pain do not have obstructive CHD (defined as lesions with ≥50% stenosis), which has been realized as a frequent issue encountered in clinical practice (Ref. 49). This clinical phenomenon is defined as coronary microvascular dysfunction (CMD), which is mainly due to capillary rarefaction and adverse remodelling of intramural coronary arterioles (Ref. 50). It has been shown that ET intervention improves microvascular structure and function (Refs. 51–53). Extensive studies with experimental animals have demonstrated that ET improves coronary capillary angiogenesis and myocardial arteriolarisation, as well as increases coronary capillary exchange capacity and maximal coronary blood flow capacity, thus promoting coronary collateral circulation growth, increasing ischemic threshold and limiting MI size (Refs. 53, 54). Additionally, activating NO, PGs and hyperpolarization factors by ET improves hemorheological parameters to reduce prothrombotic risk (Ref. 53). However, compared to preserved coronary flow reserve, patients with CMD have a higher prevalence of inducible myocardial ischemia and reduced global perfusion reserve, as well as coronary perfusion efficiency, during ET (Ref. 55). The underlying mechanism merits further investigation. Substantial evidence suggests that ET controls blood pressure throughout the day, including during rest or stressful events. ET enhances endothelium-dependent vascular relaxation through NO release and decreased ROS, and it improves extracellular matrix levels by reducing collagen deposition and matrix metallopeptidase (MMP)-2/9 expression. ET also decreases plasma levels of proinflammatory cytokines, such as TNF-α, IL-1β, and norepinephrine, and it reduces vascular injury via the NF-κB system (Refs. 52, 56). In a mouse model of MI, Chen et al. (Ref. 57) showed that lncRNA Malat1 repairs the cardiac microcirculation by mediating the miR-26b-5p/Mfn1 pathway to block effects on mitochondrial dynamics and apoptosis.

In clinical practice, invasive coronary angiography, positron emission tomography (PET), computed tomography (CT), cardiac magnetic resonance (CMR), and left ventricular contrast echocardiography are suitable for the noninvasive detection of CMD (Refs. 49, 58, 59), but these methods still need to be further tested in large-scale randomized clinical trials for intensive and individualized treatment. Therefore, more investigations should be undertaken to reveal different adaptive changes induced by different types, models, and durations of ET in different CVD populations.

### ET promotes cardiac repair and regeneration

The end-stage manifestation of many CVDs is HF, of which the pathological driver is death and loss of cardiomyocytes and supporting tissues. Although adult mammalian hearts have limited regenerative capacity, the rate of regeneration is extremely low and declines with age. There are no effective treatment strategies to supplement injured cardiomyocytes and promote cardiac regeneration. Recent advances have verified that regular ET promotes cardiac repair and regeneration by inducing physiological cardiac hypertrophy, inhibiting myocardial apoptosis and necrosis, improving cardiac metabolism, and promoting cardiomyocyte proliferation (Refs. 60, 61). Vujic et al. (Ref. 62) found that 8 weeks of voluntary running exercise significantly increased the number of new cardiomyocytes in normal adult and MI mouse hearts. Another study has also demonstrated that running exercise restores cardiomyogenesis in aged mice, which may be associated with circadian rhythm pathways (Ref. 63). ET, as a physiological stimulus, plays an important cardioprotective role in adult zebrafish by inducing cardiomyocyte proliferation (Ref. 64). Recently, animal studies have revealed that ncRNAs are linked to the control of cardiomyocyte regeneration, renewal, and proliferation. For instance, swimming or wheel exercise upregulates miR-222 expression, which targets the Kip1 (P27), homeodomain-interacting protein kinase 1/2 (HIPK1/2), and homeobox-containing 1 (HMBOX1) to induce the proliferation and growth of cardiomyocytes (Ref. 65). Other miRNAs, such as miR-342-5p (Ref. 66), miR-486 (Ref. 67), and miR-133 (Ref. 68), also play significant roles in regulating cardiac growth and survival in response to ET. Furthermore, swimming training promotes cardiomyocyte growth and attenuates cardiac remodelling in an MIRI mouse model by upregulating lncRNA CPhar expression, which inhibits the expression of transcription factor 7 (ATF7) by sequestering CCAAT/enhancer binding protein β (C/EBPβ) (Ref. 69). Although changes in ncRNA expression in animal studies are at least in part transferable to treatment regimes, more work is still needed to validate their safety and applicability for clinical application.

### ET alleviates cardiac fibrosis

Cardiac fibrosis (CF), mainly characterized by the unbalanced production and degradation of extracellular matrix (ECM) proteins, is the main pathological process of CVDs, and it leads to cardiac dysfunction, arrhythmogenesis, and adverse outcomes (Ref. 70). Attenuating CF is a key strategy for maintaining cardiac function and improving the prognosis of patients with CVDs. In addition to traditional drug therapy and new interventions, such as chimeric antigen receptor (CAR)-T-cell therapy (Refs. 71, 72), increasing evidence from clinical and animal studies suggests that ET-based CR should be taken into consideration to prevent the progression of adverse CF (Refs. 73–76). For example, ET reduces the fibrosis-related protein levels of AT_1_R, fibroblast growth factor 23 (FGF23), lysyl oxidase like-2 (LOX-2), transforming growth factor (TGF)-β, p-Smad2/3, TIMP-1/2, MMP-2/9, and collagen I (Refs. 77, 78). Mechanistically, ET suppresses LOX-2/TGF-β-mediated fibrotic pathways to prevent CF and myocardial abnormalities in early-aged hypertension (Ref. 77). In addition, ET increases FGF21 protein expression and regulates the TGF-β1-smad2/3-MMP2/9 axis (Ref. 79). ET markedly inhibits lncRNA MIAT expression and upregulates miR-150 to improve cardiac remodeling by inhibiting P2X7 purinergic receptors (P2X7Rs) in diabetic cardiomyopathy (DCM) (Ref. 80). Additionally, ET plays a functional role in HF and DOX-induced cardiotoxicity (Refs. 78, 81, 82). Although basic and clinical trials associated with antifibrotic drugs have been performed, future studies should focus on exploring the underlying pathophysiological mechanisms in the onset and progression of CF to determine integrated and personalized therapeutic strategies. In summary, the early identification, diagnosis, and management of CF are vital in improving the survival and prognosis of CVD patients.

### Exercise mediates cardiac protection by ncRNAs

Most recently, an increasing number of studies have indicated that epigenetic modifications are involved in the promotion of cardiac health and the prevention of CVDs. Lifestyle factors, such as exercise and diet, extensively induce epigenetic modifications, including DNA/RNA methylation, histone posttranslational modifications, and ncRNAs (Refs. 83, 84). For example, a low-protein diet causes altered sncRNA content in spermatozoa, which is associated with altered levels of lipid metabolites in offspring and decreased expression of specific genes starting in two-cell embryos (Ref. 85). However, no reported studies have evaluated the link of exercise-induced DNA methylation in cardiac tissue, indicating that the action of different epigenetic mechanisms in EIC needs further study.

**Figure 2.**
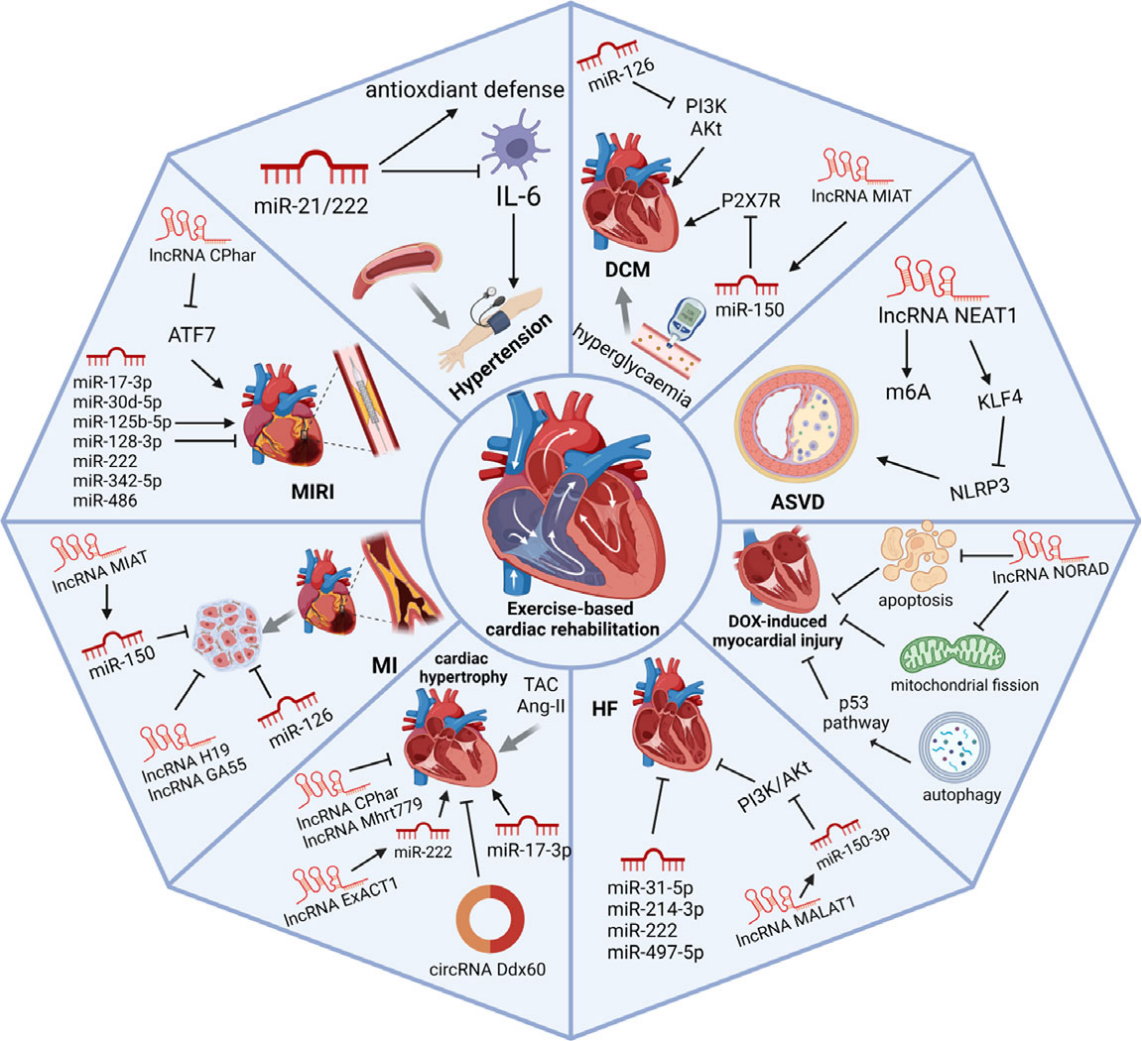
Exercise-induced ncRNAs and their regulated pathways in CVDs. The regulation of ncRNAs contributes to the progression of various CVDs, including hypertension, DCM, ASVDs, MIRI, MI, HF, and DOX-induced cardiomyopathy.

Evidence suggests that ncRNAs may be used as novel biomarkers, offering innovative prospects for the diagnosis, treatment, and prognosis of CVDs. In addition to the changes resulting from pathological conditions, ncRNAs and related signalling pathways also undergo changes due to ET (Figure 2). For example, miR-1-3p is an emerging biomarker of high-volume maximal endurance exercise, while in low-volume doses there is an absence of response in low-volume doses (Ref. 98). CircRNA MBOAT2 expression is significantly decreased after 24 h of marathon running and can be used as a biomarker for detecting cardiopulmonary adaptation (Ref. 96). There are many studies of miRNA involvement in exercise adaptations, and far less is currently known about lncRNAs and circRNAs (Table 1). NcRNAs and their signal pathways respond differently to exercise intensity, frequency, and tolerance. Additionally, the correlation between exercise prescription and ncRNAs needs to be further researched in both animal models and clinical cohort studies.

**Table 1.** Exercise mediates ncRNAs in cardiovascular diseases

ncRNAs and expression	Targets and expression	Disease	Experimentalphenotype	Exercise			Functional role	Reference
				Type	intensity	Duration		
miR–17–3p^#^↑	TIMP–3*↓, PTEN*↓	MIRI, TAC	C57BL/6 J mice, NRCMs	Ramp swimming, voluntary wheel exercise	NA	3 weeks	Enhance cardiomyocyte proliferation, pro-hypertrophy	(86)
miR–21/222↓	TAC↑, MDA, IL–6↓	Hypertension	Patient	Cycle ergometer	Moderate	3d/week, 6 weeks	Anti-inflammation pro-antioxidant defence	(30)
miR–126*↑	PIK3↓, Akt, eNOS, MAPK	MI, DCM	SD rat, HUVECs	Motorized rodent treadmill	Moderate and high	5d/week, 4–8 weeks	Pro-angiogenesis anti-cardiac remodeling	Song et al. (87; 88)
miR–30d–5p↑ miR–125b–5p↑, miR–128–3p↑,	MAPK↓	MIRI	C57BL/6 J mice	Swimming	NA	4 weeks	Anti-apoptosis	(89)
miR–31a–5p↑ miR–214–3p↑ miR–497–5p↑	NA	HF	SD rat, hiPSC-CMs	Uphill running	Moderate and high	5d/week, 6–8 weeks	Anti-fibrosis reduced Ca^2+^ decay	(90)
miR–222^#^↑	P27*↓, HMBOX1*↓, HIPK1/2*↓	MIRI, HF	patient, C57BL/6 J mice, NRVMs	Ramp swimming, voluntary wheel exercise	NA	3 weeks	Anti-cardiac remodeling and dysfunction	(65)
exo-miR–342–5p^#^↑	Caspase 9↓, JNK 2↓	MIRI	SD rat, patient, NRVMs	Swimming	high	7d/week, 4 weeks	Anti-apoptosis pro-survival	(66)
miR–486^#, *^↑	PTEN*↓, FoxO1*↓, Akt/mTOR	MIRI	C57BL/6 J mice, NRCMs, NRCFs, hiPSC-CMs	Swimming	NA	twice/d, 4 weeks	Anti-apoptosis	(67)
lncRNA NEAT1^#^↓	m6A↓, KLF4^#^↑, NLRP3↓	ASVD	C57BL/6 J mice, patient	Treadmill training	Moderate	5d/week, 12 weeks	Mitigate endothelial pyroptosis	(91)
lncRNA MIAT↓	miR–150↑, P2X7R^#^↓	MI, DCM	C57BL/6 J mice	Treadmill training	Low	5d/week, 12 weeks	Anti-cardiac remodeling	Farsangi et al. (73); Wang et al. (80)
lncRNA H19↑, lncRNA GA55↑	NA	MI	Wistar rat	Motor-drive treadmill	low	5d/week, 4 weeks	Anti-fibrosis	(73)
lncRNA CPhar^#^↑	ATF7*↓, C/EBPβ*↓	MIRI, hypertrophy	C57BL/6 J mice, NMCMs	Swimming	NA	3 weeks	Pro-myocardial cell growth, anti-fibrosis	(69)
lncRNA MALAT1^#, *^↓	miR–150–3p*↑ PI3K/Akt↓	HF	Wistar rat	Treadmill training	moderate	5d/week, 8 weeks	Anti-fibrosis and apoptosis, pro-autophagy	(92)
lncRNA ExACT1*	miR–222, DCHS2^#^, Yap1	TAC-HF, hypertrophy	Mice, patient zebrafish, cardiomyocyte	Voluntary exercise	NA	8 weeks	Induced-pathological and physiological hypertrophy, pro-cardiomyogenesis	(93)
lncRNA Mhrt779^#^↑	Brg1, Hdac2, p-Akt, p-GSK3β	Hypertrophy	C57BL/6 J mice, cardiomyocyte	Swimming	NA	4 weeks	Anti-cardiac hypertrophy	(94)
circ Ddx60↑	eEF2, AMPK	Hypertrophy	C57BL/6 J mice	Swimming	NA	4 weeks	anti-cardiac hypertrophy	(95)
circRNA MBOAT2↓	NA	Health	Volunteer	Marathon			Biomarker of cardiopulmonary adaptation	(96)
miR–103a–3p	NA	Health	Volunteer	10-km race, a half- marathon and marathon			Decrease SBP, improve cardiac structure and function	(97)
miR–33a–5p miR–505–3p miR–345–5p miR–424–3p miR–1260a	NA	Health	Volunteer	10-km race, a half- marathon and marathon			Exercise dose biomarkers	(98)

*Notes*: ↑ represents ‘enhanced effect’; ↓ represents ‘reduced effect’; NA represents ‘not available’; *represents ‘cell transfection’; ^#^represents ‘genetic animal model’.

### Exercise-induced ncRNAs in MI

MI is a leading cause of cardiovascular death and chronic HF worldwide (Ref. 1). Cardiomyocytes undergo a series of pathological adaptations after MI, including myocardial ischemia, hypoxia, inflammatory response, necrosis, progressive CF, and ventricular enlargement. Persisting structural cardiac abnormalities are associated with arrhythmias, HF, and sudden cardiac death. Although percutaneous coronary intervention and drugs have become the predominant treatment for MI, these strategies cannot reverse or attenuate the biological process, eliminate risk factors, and consistently improve patient outcomes.

It has been demonstrated that regular ET significantly improves cardiac structure and function by rescuing post-MI stunned myocardium (Refs. 4, 9). Furthermore, exercise-mediated ncRNAs have great significance in the biological regulation of MI. in vivo studies have shown that 4 weeks of ET increases miR-126 expression and reduces the expression of PIK3R2 and SPRED1. in vitro results have demonstrated that miR-126 promotes angiogenesis by the PI3K/Akt/eNOS and MAPK signalling pathways, subsequently improving of MI cardiac function, including increasing left ventricular systolic pressure (LVSP) and + dp/dt_max_ and decreasing left ventricular end-diastolic pressure (LVEDP) and collagen volume fraction (Ref. 87). Compared to the control group, ET reduces cardiomyocyte apoptosis and improves CF and systolic function by decreasing lncRNA MIAT expression and increasing the expression of lncRNA H19 and lncRNA GA55 (Ref. 73). Further exploration has shown that overexpression of lncRNA H19 also dramatically alleviates myocardial infarct size and inflammation by sponging miR-22-3p to target lysine (K)-specific demethylase 3A (KDM3A) (Ref. 99). Likewise, other studies have illustrated that lncRNA H19 plays an essential role in regulating the pathological processes of CVDs by acting as a molecular sponge or interacting with various proteins to target gene expression (Ref. 100). The above results indicate that lncRNA H19 is a potential marker or a promising target for CVD treatment.

Clinical outcomes and prognosis vary with individual differences in pathophysiological mechanisms. In response to the common cardiac remodelling in the early and late stages of MI, there are currently novel therapeutic strategies, including SGLT2-i, inflammatory modulators, and silencing small RNAs, have been developed (Ref. 72). Furthermore, circulating markers can be combined with novel cardiac imaging techniques, such as CMR and myocardial work index of the left ventricular 17 segment by echocardiography, to help reveal the pathophysiological mechanism of cardiomyopathy and better guide personalized treatment.

### Exercise-induced ncRNAs in myocardial ischemia-reperfusion injury

Recovering reperfusion after MI can cause irreversible detrimental effects known as MIRI, including myocardial stunning, reperfusion arrhythmia, no-reflow phenomenon, and lethal reperfusion injury. Pathological changes, such as inflammation, apoptosis, autophagy, and neurohumoral activation, are considered to have the same underlying cause as MIRI (Ref. 101). Recent findings have revealed that novel ncRNAs are involved in a variety of important biological processes and the development of MIRI (Ref. 102). Systemic reviews and meta-analyses have also shown that ET increases the left ventricular ejection fraction, cardiac output, and coronary blood flow (Ref. 103). Increased miR-17-3p inhibits TIMP 3 expression to enhance cardiomyocyte proliferation by activating EGFR/JNK/SP-1 signalling (Ref. 86). In addition, miR-17-3p indirectly regulates the PTEN/Akt signalling pathway to promote cardiomyocyte hypertrophy in vivo and in vitro (Ref. 86). Bei et al. reported that downregulation of miR-486 occurs in both MIRI in vivo and OGDR-treated cardiomyocytes in vitro. However, AAV9-mediated miR-486 overexpression in a mouse model of MIRI significantly reduces infarct size, the Bax/Bcl-2 ratio, and caspase-3 cleavage (Ref. 67). Functionally, increasing miR-486 is protective against MIRI and myocardial apoptosis through activating the Akt/mTOR pathway to inhibit PTEN and FoxO1 expression (Ref. 67). Concurrently, Hou et al. revealed a novel endogenous cardioprotective mechanism in which long-term exercise-derived circulating exosomal miR-342-5p protects the heart against MIRI. Mechanistically, miR-342-5p targets caspase 9 and JNK 2 to inhibit hypoxia/reoxygenation-induced cardiomyocyte apoptosis; it also enhances survival signalling (p-Akt) by targeting phosphatase gene Ppm1f (Ref. 66). Other miRNAs, such as miR-125-5p, miR-128-3p, and miR-30d-5p, are also important regulators of EIC against MIRI (Ref. 89). However, there are still few reports about exercise-mediated other ncRNAs (lncRNAs and circRNAs) in MIRI. Therefore, further studies are needed to clarify the underlying mechanisms of exercise-mediated ncRNAs in the occurrence and development of MIRI.

### Exercise-induced ncRNAs in HF

Exercise-based CR has been recommended as a clinical consultation for HF by international guidelines. Individualized exercise prescription is effective in improving the 6-minute walk distance, cardiopulmonary function, incidence of complications, and quality of life (Refs. 104, 105). Emerging ncRNAs have been found to play roles in EIC and HF (Ref. 106). For instance, Stølen et al. (Ref. 90) found that moderate- and high-intensity ET decrease the expression of miR-31a-5p, miR-214-3p, and miR-495-5P in post-MI HF mice, which reduces arrhythmia susceptibility by slowing Ca^2+^ transient decay and decreasing collagen content, such as CTGF, collagen 1α1, and TGFβ1 expression. Other ncRNAs, such as miR-1, miR-133, miR-15 family, and circRNA-010567, inhibit myocardial fibrosis by regulating related pathways, including Ang-II, MAPKs, and TGF-β (Refs. 106, 107). However, the number of exercise-induced lncRNAs and circRNAs is limited, and their roles in EIC for HF are unclear. As reported by Hu et al. (Ref. 92), aerobic exercise improves left ventricular remodelling and cardiac function by inhibiting lncRNA MALAT1 to regulate the miR-150-5p/PI3K/Akt signalling pathway. Overexpression of lncRNA ExACT1 has been shown to aggravate pathological hypertrophy and HF, while inhibition of lncRNA ExACT1 protects against CF and dysfunction by inducing physiological hypertrophy and cardiomyogenesis (Ref. 93). The potential regulatory mechanism is that the function of lncRNA ExACT1 is regulated by miR-222, calcineurin signalling, and Hippo/Yap1 signalling through dachsous cadherin-related 2 (DCHS2) (Ref. 93), which provides a potentially tractable therapeutic target for EIC in HF.

### Exercise-induced ncRNAs in cardiac hypertrophy

Cardiac hypertrophy is characterized by a marked increase in myocardial mass index, and it can be categorized as physiological or pathological hypertrophy. Physiological myocardial hypertrophy (PMH) is an adaptive and reversible cardiac growth under chronic exercise stimulation and exerts cardioprotective effects. Conversely, pathological hypertrophy develops in response to chronic pressure or volume overload in disease settings, such as transverse aortic constriction (TAC) and aortic stenosis, resulting in adverse cardiac remodelling and dysfunction. Evidence suggests that ncRNAs are directly involved in and regulate different pathological stresses of cardiac hypertrophy. For instance, knocking down or overexpression of miR-30d and certain lncRNAs (Chast, Chaer, NRON, Mhrt, and H19) plays important regulatory roles in a mouse model of TAC-induced cardiac hypertrophy (Refs. 108, 109).

The roles of ncRNAs in exercise-induced cardiac hypertrophy are supported by findings in different animal models and training programs. The expression of miR-21, miR-27a, and miR-143 is different after aerobic swimming training in mice presenting physiological left ventricular hypertrophy compared with sedentary controls (Ref. 97). Moreover, silencing lncRNA Mhrt779 attenuates the antihypertrophic effect of exercise hypertrophic preconditioning (EHP) in TAC mice and in cultured cardiomyocytes treated with Ang-II, while overexpression of lncRNA Mhrt779 enhances the antihypertrophic effect. Mechanistically, ET increases resistance to pathological pressure overload by an antihypertrophic effect mediated by the lncRNA Mhrt779/Brg1/Hdac2/p-Akt/p-GSK3β signalling pathway (Ref. 94). Similarly, lncRNA CPhar and lncRNA ExACT1 have emerged as important mediators underpinning the process of exercise-induced PMH (Refs. 69, 93). Although some circRNAs, such as circRNA sh3rfe (Ref. 110), circRNA Cacna1c (Ref. 111), circRNA 0001052 (Ref. 112), and circRNA Ddx60 (Ref. 95), have been found to be closely related to cardiac hypertrophy, only circRNA Ddx60 has been identified to be needed for exercise-induced PMH in mice on the basis of a forced swim training model. CircRNA Ddx60 contributes to the antihypertrophic effect of EHP by binding and activating eukaryotic elongation factor 2 (eEF2) (Ref. 95).

Notably, multiple factors, such as genetic background, species, and gender differences, may influence the adaptations and outcomes of exercise. Konhilas et al. (Ref. 113) found that female mice have a greater increase in PMH after treadmill or voluntary wheel running. It has been revealed that common genetic variants are important pathogenic factors in hypertrophic cardiomyopathy, suggesting the existence of non-Mendelian inheritance patterns with ethnic differences (Ref. 114). Current knowledge on the different expression, regulation, and pathology-related functions of ncRNAs in terms of both sex and age is limited (Refs. 115, 116). Additionally, whether genetic or sex differences influence the expression of ET-induced ncRNAs and their signalling pathway responses remain to be clarified.

### Exercise-induced ncRNAs in other CVDs

T2DM is a metabolic disorder characterized by hyperglycaemia, hyperinsulinemia and high insulin resistance, which causes macrovascular and microvascular complications, such as atherosclerosis, CHD, and diabetic cardiomyopathy (DCM). Increasing ncRNAs have been recommended as exercise indicators for cardiovascular prescriptions and as preventive or therapeutic targets for cardiovascular complications in T2DM. MiR-126 induced by ET has been found to decrease vascular inflammation and apoptosis via the PI3K/Akt pathway, promote angiogenesis via the VEGF pathway, and increase cardiac autophagy via the PI3K/Akt/mTOR pathway (Refs. 88, 117). Long-term ET protects against vascular endothelial injury of insulin resistance by downregulating the expression of several lncRNAs, including FR030200 and FR402720 (Ref. 118) and then attenuating the progression of atherosclerotic CVD. Similarly, lncRNA NEAT1 induces endothelial pyroptosis by binding Kruppel-like factor 4 (KLF4) to promote the transcriptional activation of the key pyroptotic protein, NOD-like receptor thermal protein domain-associated protein 3 (NLRP3), whereas exercise reverses these effects (Ref. 91).

In addition, ncRNAs play significant roles in other CVDs, such as valvular heart diseases (Ref. 119), cardiomyopathy (Ref. 120), myocarditis (Ref. 121), and pulmonary hypertension (Ref. 122). The main pathophysiological processes also include inflammation, oxidative stress, apoptosis, extracellular matrix reorganization, and fibrosis, but they are still not sufficiently understood in terms of biological mechanisms. Although an increasing number of studies and guidelines have demonstrated that exercise-based CR has beneficial effects on these CVDs (Refs. 10, 123, 124), the specific molecular mechanisms and signaling pathways of exercise-induced ncRNAs await further investigation. Consequently, this limits improvements in novel therapeutic strategies and biomarkers of risk assessment, as well as prevention and diagnosis of CVDs. In addition, due to significant differences in cardiopulmonary function and different recovery processes after CVD events, it is necessary to develop an optimal individualized exercise-based CR program oriented to clinical problems according to the condition of patients.

## Conclusions and perspectives

Despite the benefits of exercise and ncRNAs in CVDs, there is a limited understanding of the molecular regulatory mechanisms of exercise-induced ncRNAs. Additionally, performing exercise-based CR and detecting cardiac biomarkers in clinical practice have been challenging. Exploring the pathophysiological roles and molecular mechanisms induced by physical exercise can facilitate potential alternative strategies for CVD prevention and treatment, as well as facilitate the development of personalized exercise prescriptions. However, several fundamental problems hinder the clinical application of ncRNAs as novel biomarkers and therapeutic targets.

It is challenging to compare ncRNAs and exercise in published studies. First, disputes exist as to whether different exercise prescriptions (including type, intensity, frequency, duration, volume, and progression) benefit CVDs. For instance, recent studies have shown that moderate-intensity continuous training (MICT) has a positive effect on the cardiopulmonary function and physical performance of patients after transcatheter aortic valve replacement (TAVR) (Refs. 10, 125), whereas few studies have developed the application of high-intensity interval training (HIIT) in patients after TAVR. Thus, the HIIT-induced mechanism remains unclear.

NcRNAs show significant differences in expression during early, middle, and long-term ET, as well as between chronic and acute endurance ET. Studies have revealed that miR-1 and miR-133a expression is elevated following one-time resistance exercise but downregulated following long-term endurance ET. Furthermore, the expression level of ncRNAs may change dramatically during pathological processes of CVDs. For instance, lncRNA H19 expression is markedly upregulated post-MI at the infarct border zone with a peak during four to seven days and subsequently decreases within the following three weeks (Refs. 73, 126). Therefore, the time course of exercise-induced ncRNAs in circulation needs to be considered and clinical indicators, such as cardiac troponin, electrocardiogram, and CMR, need to be combined to explore their predictive value in different stages of CVD development.

Human studies investigating ncRNAs are far from perfect. There are significant differences in ncRNA expression in serum and plasma, which requires more precise approaches to detect ncRNAs and to combine more omics, such as metabolomics, to analyse the molecular mechanism of CVDs. Thottakara et al. (Ref. 127) reported the first evidence that miR-4454 expression is markedly increased in the plasma of hypertrophic cardiomyopathy patients compared to healthy individuals and that elevated miR-4454 levels are associated with the severity of CF, which is detected by CMR, suggesting that miR-4454 may be a potential biomarker of fibrosis. Compared to basic experiments, there are more confounding factors in clinical work, including study design, gender, age, lifestyle, chronic diseases, individual differences, and medical intervention. It is difficult to determine whether these confounding factors have uncertain effects on ncRNA expression in the heart.

Above all, exercise-induced ncRNAs in these preliminary studies have provided a positive perspective on the pathophysiological regulation of CVDs. However, other ncRNA families, particularly circRNAs, remain to be further explored in both exercise-based CR and pathological models. Consequently, elucidating the molecular mechanisms involved in exercise-mediated ncRNAs, diseases, and health will help to discover novel biomarkers, as well as therapeutic strategies and improve quality of life.

## Abbreviations

ASVD, atherosclerosis vascular disease; ATF7, activating transcription factor 7; BCAAs, branched-chain amino acids; C/EBPβ, CCAAT/enhancer binding protein β; CF, cardiac fibrosis; CHD, coronary heart disease; CMD, coronary microvascular dysfunction; CMR, cardiac magnetic resonance; CR, cardiac rehabilitation; CRP, C-reactive protein; CT, computed tomography; CVD, cardiovascular disease; DCM, diabetic cardiomyopathy; DCHS2, dachsous cadherin-related 2; ECM, extracellular matrix; eEF2, eukaryotic elongation factor 2; EHP, exercise hypertrophic preconditioning; EIC, exercise-induced cardioprotection; ET, exercise training; FGF21, fibroblast growth factor 21; FRAP, ferric reducing antioxidant power; GSH-Px, glutathione peroxidase; HF, heart failure; HMBOX1, homeobox-containing 1; HO-1, heme oxygenase 1; HIIT, high-intensity interval training; HIPK1/2, homeodomain-interacting protein kinase 1/2; IL-1, interleukin-1; KDM3A, lysine (K)-specific demethylase 3A; KLF4, Kruppel-like factor 4; LVSP, left ventricular systolic pressure; LOX-2, lysy1 oxidase like-2; LVEDP, left ventricular end-diastolic pressure; MICT, moderate-intensity continuous training; MIRI, myocardial ischemia–reperfusion injury; MMP, matrix metallopeptidase; MI, myocardial infarction; MKL1, megakaryocytic leukaemia 1; ncRNA, noncoding RNA; NF-κB, nuclear factor-κB; NO, nitric oxide; NRF2, nuclear factor erythroid 2-related factor; NLRP3, NOD-like receptor thermal protein domain-associated protein 3; P2X7R, P2X7 purinergic receptors; PET, positron emission tomography; PMH, physiological myocardial hypertrophy; ROS, reactive oxygen species; SOD, superoxide dismutase; SIRT1, sirtuin 1; T2DM, type 2 diabetes mellitus; TAC, transverse aortic constriction; TAVR, transcatheter aortic valve replacement; TGF-β, transforming growth factor-β; TNF-α, tumour necrosis factor-α; VCAM1, vascular cell adhesion molecule-1; VSMC, vascular smooth muscle cell; vWF, von Willebrand factor.
